# Expert Testimony

**Published:** 1893-02

**Authors:** 


### EXPERT TESTIMONY.

A case was recently decided by Judge Baker, of the Superior Court in this city, which is of much interest to physicians, who may be called upon to render professional expert service in the trial of a case in court, in relation to the collection of fees for such service.

The facts as testified were essentially as follows: The father-in-law of an insane gentleman, whose insanity rendered restraint necessary and at the same time exceedingly difficult of legal application, retained a lawyer to attempt to have his son-in-law declared insane. This lawyer undertook to conduct the case, with the understanding that he should be allowed to employ two particular experts, in whose skill he had confidence.

The experts were engaged by the lawyer, but nothing was said about fees further than that while the father-in-law was able to pay a full fee for such services he felt that his relation to the case was not so vital as to bind him very strongly, and he would therefore expect considerable concessions in the matter of fees from all parties concerned.

The expert who brought suit showed that he had prepared a paper presenting his views of the case and that he read this paper or parts of it in the presence of the father-in-law and his counsel; that the father-in-law praised the production and understood the purpose of it and that it was used in court as intended. This the expert in his suit to collect his fees held was equivalent to a contract with the defendant, for he allowed the expert to proceed in his behalf, knowing definitely the nature of the service being rendered.

His honor held that the plaintiff had not made a contract for the services of the expert and was not therefore bound to pay him. Nor was the plaintiff bound by the contract of his attorney.

The only safe plan for a physician under such circumstances would appear to be to make a definite contract duly witnessed with the parties receiving the service, and with no one else, otherwise he is exceedingly liable to be "lawed" out of his fees. —*North American Practitioner.*

